# Sporadic and rapidly progressive arrhythmogenic right ventricular cardiomyopathy in a 12-year-old boy who was diagnosed with epilepsy

**DOI:** 10.1093/omcr/omab046

**Published:** 2021-06-18

**Authors:** Alaa Al-Khleaf, Amal Babi, Mulham Jarjanazi, Walid Haddad

**Affiliations:** 1 Department of Internal Medicine, Faculty of Medicine, Aleppo University Hospital and Aleppo University Hospital of Heart, University of Aleppo, Syria; 2 Faculty of Medicine, University of Aleppo, Syria

**Keywords:** ARVC, cardiomyopathy, case report

## Abstract

Arrhythmogenic right ventricular cardiomyopathy (ARVC) is one of the leading causes of sudden cardiac death amongst young people and athletes. In this genetic disease, arrhythmia and fibro-fatty changes in the right ventricular myocardium are the main characteristics of the disease.

Here, we report a case of ARVC in a 12-year-old boy who was previously diagnosed with epilepsy, the patient’s condition manifested sporadically and was complicated by rapid progression, and unfortunate fatal deterioration after admission into the pediatric emergency room due to fatigue, dizziness and palpitation.

A diagnosis of ARVC was established, even though a family history was absent. Due to possible rapid deterioration, as described in this case, we recommend immediate primary and secondary prevention of arrhythmias in these patients, and to take in consideration of the potential risks of using sodium valproate in these patients.

## INTRODUCTION

Arrhythmogenic right ventricular cardiomyopathy (ARVC) or arrhythmogenic right ventricular dysplasia (ARVD) is an inherited—in most of the cases—genetic heart muscle disorder characterized by arrhythmia and fibro-fatty changes in the heart wall, with estimated prevalence of 1:2500 to 1:5000 in both sporadic/familial cases, with male predominance.

The common genetic causes known to be associated with ARVC are DSC2, DSG2, DSP, JUP, PKP2 and TMEM43. Less common genetic causes include CTNNA3, DES, LMNA, PLN, RYR2, TGFB3 and TTN. A subset of these 13 genes encode components of the desmosome in addition to the known genetic loci that have been associated with ARVC are 14q23-q24 (ARVC1), 1q42-q43 (ARVC2), 14q12-q22 (ARVC3), 2q32 (ARVC4), 3p25 (ARVC5), 10p12-p14 (ARVC6), 10q22, 6p24 (ARVC8) and 12p11 (ARVC9).

The main symptoms of ARVC are palpitations, syncope, atypical chest pain, dyspnea and right heart failure. Its prevalence is estimated to be 1 in 2000 to 1 in 5000 of the population. ARVC is a disorder that is often diagnosed between the ages of 10 and 50 with a mean age of 30 [1–6].

Here, we present a case of a young male who was first diagnosed with epilepsy and was treated accordingly. A diagnosis of ARVC was established, even though a family history was absent.

## CASE REPORT

A 12-year-old Syrian male was admitted to the pediatric emergency room with fatigue, dizziness and palpitation. A previous neurological examination led to the diagnosis of epilepsy as the child manifested recurrent seizures. Also, we performed EEG that revealed centro-parietal theta rhythmic activity with 2–3 hz generalized waves and spikes, which is a characteristic EEG pattern in infants and children with primary generalized seizures.

However, the child did not exhibit any particular subtype of epilepsy, and since the first incident of seizures, the child took sodium valproate daily with a dosage of 500 mg repeated twice.

On physical examination, the patient was responding well to the examiner. His blood pressure measurement was 90 over 60. His pulse was feeble and rapid. Electrocardiography (ECG) showed sustained wide QRS tachycardia (ventricular tachycardia) that mimics a left bundle branch block (LBBB) morphology in the superior axis, that is, negative QRS in leads II, III and AVF, and positive in lead AVL. The patient was initially given Amiodarone with a loading dose of 5 mg/kg over 20 to 60 minutes repeated twice up to a maximum dose of 15 mg/kg. The patient did not respond well to the initial treatment. Synchronized direct-current (DC) was carried out, which successfully restored sinus rhythm.

Following hemodynamic stability, biochemical testing was performed. Complete blood count (CBC), K+, Ca2+, Mg2+, Na+, Cr and TSH values were normal. On the other hand, 12-lead resting ECG showed multiple abnormalities, including right bundle branch block (RBBB), T-wave inversion and Epsilon waves on precordial leads V1 up to V5. Transthoracic echocardiography (TTE) revealed structural alteration of the right ventricle (RV) and blood stagnation. More specifically, the parasternal short-axis (PSAX) right ventricular outflow tract (RVOT) value was 35 mm, along with regional dyskinesia ofRV.

**Figure 1:**
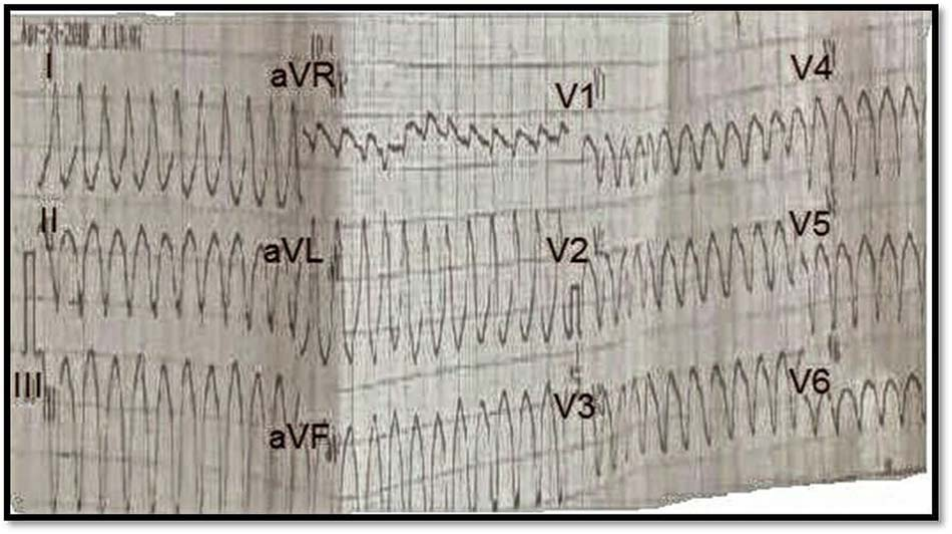
ECG on presentation, showing sustained wide QRS tachycardia (VT) that mimic LBBB morphology in superioraxis.

**Figure 2:**
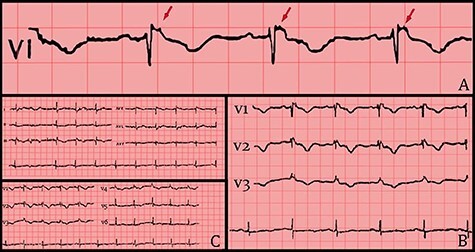
(A) 12 leads ECG after hemodynamic stability. (B) ECG shows RBBB morphology, T-wave inversion and Epsilon waves on precordial leads. (C) Epsilon waves on lead V1 (red arrows).

Afterward, the patient was discharged on Amiodarone and Bisoprolol, along with regular monitoring, which revealed progressive structural alteration of the right ventricle on TTE and atrial fibrillation (AF) on ECG (Fig. 1) (Fig. 2).

**Figure 3:**
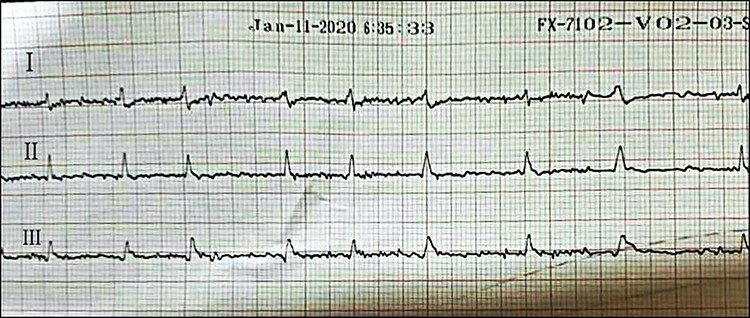
ECG showing atrial fibrillation during follow-up.

**Figure 4:**
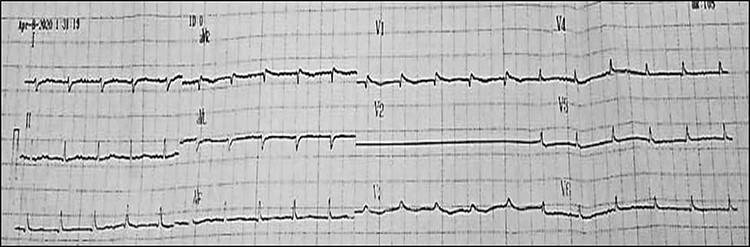
Last hospitalization, ECG showingAF.

**Figure 5:**
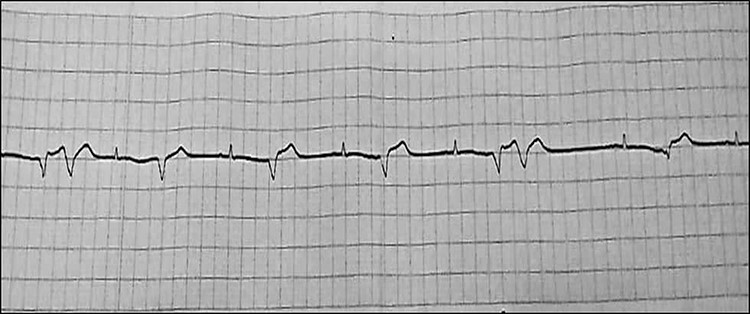
Last hospitalization, ECG showing AF withPVCs.

**Figure 6:**
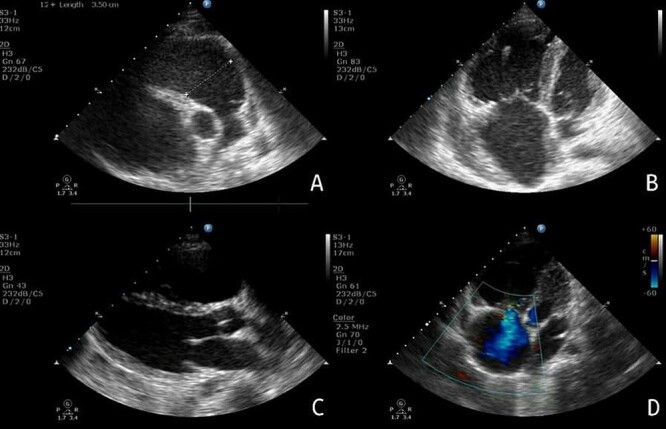
Echocardiography: (A) PSAX view, showing the extent of RV enlargement (PSAX RVOT = 35 mm). (B) Apical view of the four chambers, showing enlargement of the right-side chambers. (C) PLAX view. (D) Apical view of the four chambers, showing tricuspid valve insufficiency.

After one year of follow-up, the patient was admitted to the pediatric emergency with fatigue, dizziness and cyanosis. On physical examination, the patient’s systolic/diastolic blood pressure measurement was 80 to 60. ECG revealed rapid atrial fibrillation with premature ventricular contractions (PVCs) (Figs 3 and 4). TTE showed extreme enlargement of the right chambers along with significant tricuspid regurgitation. PSAX RVOT value was 53 mm. The patient was hospitalized. However, he rapidly succumbed to death due to severe right ventricular insufficiency. Postmortem myocardial biopsy was not performed because it was against the family wishes (Figs 5 and 6).

## DISCUSSION

ARVC is an inherited cardiomyopathy that affects a significant number of first-degree relatives of the proband. However, many patients with ARVC remain clinically silent and asymptomatic for decades, making the disease difficult to recognize, especially in sporadic cases with no documented familial involvement. Moreover, ARVC is characterized by a clinical presentation that includes documented arrhythmia and myocardial structural abnormalities. Meanwhile, the progression of the disease over time and predictors of arrhythmias are still to be defined.

Here, we report a sporadic case of ARVC in a 12-year-old boy who was previously diagnosed with epilepsy. However, there was no documented familial involvement. Very few cases in the literature mention mutations in apparently sporadic ARVC patients whose parents did not exhibit genetic mutations or cardiac manifestations [7, 8]. In our case and due to the ongoing crisis in Syria, neither the researchers nor the patient had access to genetic testing. Therefore, to screen the family, we were left with performing regular ECG and TTE on the parents and siblings, which showed normal findings.

The diagnostic process involved generating a differential diagnosis to rule out the common causes of palpitations. Blood tests revealed no signs of hyperthyroidism or ion disturbances, which could have caused palpitations. Moreover, the absence of infection and associated symptoms in lab findings and in child’s medical history ruled out myocarditis and Chagas disease. Furthermore, there was no evidence of extracardiac symptoms like lymphadenopathy, shortness of breath or dry, hacking cough and considering the rarity of childhood sarcoidosis, which is most frequently diagnosed between 20 and 29 years of age, we did not consider sarcoidosis as a potential cause. Also, the normal findings on chest X-ray and the absence of any signs of infection in the child or his family members made cardiac tuberculosis unlikely. However, we could not perform a cardiac biopsy as a confirmatory test due to both technical and financial reasons. Furthermore, postmortem myocardial biopsy was not performed as it was against the family’s wishes, which made the exclusion of sarcoidosis and tuberculosis ultimately impossible.

ECG findings ruled out Wolff-Parkinson-White (WPW) syndrome, supraventricular tachycardia (SVT), long QT syndrome (LQTS), and Brugada syndrome. Furthermore, characteristic ECG and TTE findings led us to consider a diagnosis of ARVC according to the modified Task Force Criteria [9]. Correspondingly, the case presentation satisfied three major and one minor criteria. More specifically, sustained ventricular tachycardia (VT) mimicking LBBB morphology in superior axis, T-wave inversion that spanned the precordial leads, Epsilon waves on precordial leads and PSAX RVOT ≥32 to <36 mm along with RV dyskinesia on TTE were all recognized. Therefore, we decided on a diagnosis ofARVC.

An implantable cardioverter-defibrillator (ICD) implantation was planned due to the progressive structural alteration of RV, as was reflected by TTE and ECG findings during follow-up. However, the rapid progression of the disease from the first onset of symptoms to severe right heart failure over the course of only one year, along with the surrounding circumstances in Syria imposed by the ongoing crisis, made an ICD implantation infeasible, for both financial and logistic reasons.

Finally, considering that cardiovascular side effects of sodium valproate are reported in 1–5% of the patients, which include hypertension, tachycardia and palpitations, we think that sodium valproate could have introduced potential risks in this patient [10].

To our knowledge, this is a rare case of ARVC in which the condition manifested sporadically and was complicated by rapid progression, and unfortunate fatal deterioration, in a patient who was previously diagnosed with epilepsy. Thus, we recommend immediate primary and secondary prevention of arrhythmias in ARVC patients, and to take in consideration of the potential risks of using sodium valproate in these patients.
